# Long-term trials of colchicine for secondary prevention of vascular events: a meta-analysis

**DOI:** 10.1093/eurheartj/ehaf174

**Published:** 2025-05-02

**Authors:** Michelle Samuel, Colin Berry, Marie-Pierre Dubé, Wolfgang Koenig, José López-Sendón, Aldo Pietro Maggioni, Fausto J Pinto, François Roubille, Jean-Claude Tardif

**Affiliations:** University Medical Center Groningen, University of Groningen, Groningen, The Netherlands; Dalhousie University, Halifax, Canada; School of Cardiovascular and Metabolic Health, University of Glasgow and Golden Jubilee National Hospital, Clydebank, UK; Montreal Heart Institute, Université de Montréal, 5000 Belanger Street, Montreal, QC H1T 1C8, Canada; Technical University of Munich, School of Medicine and Health, German Heart Centre, TUM University Hospital, Munich, Germany; German Centre for Cardiovascular Research (DZHK), partner site Munich Heart Alliance, Munich, Germany; Institute of Epidemiology and Medical Biometry, University of Ulm, Ulm, Germany; IdiPaz Research Institute, Hospital La Paz, Universidad Autonoma de Madrid, Madrid, Spain; ANMCO Research Center-Heart Care Foundation, Florence, Italy; Santa Maria University Hospital, ULSSM, Center of Cardiology of the University of Lisbon, Lisbon School of Medicine, Lisbon Academic Medical Center, Lisboa, Portugal; PhyMedExp, Cardiology Department, University of Montpellier, INSERM, CNRS UMR, INI-CRT, Montpellier, France; Montreal Heart Institute, Université de Montréal, 5000 Belanger Street, Montreal, QC H1T 1C8, Canada

**Keywords:** Colchicine, Coronary artery disease, Atherosclerosis, Cardiovascular disease, Myocardial infarction, Stroke, Secondary prevention, Inflammation

## Abstract

**Background and Aims:**

Colchicine has emerged as a safe and inexpensive anti-inflammatory medication to target the residual risk of cardiovascular events in the secondary prevention of coronary artery disease. Two recently published randomized controlled trials (RCTs) investigating colchicine in the post-stroke and post-myocardial infarction (MI) populations warrant a re-evaluation of colchicine. New evidence was synthesized in a systematic review and meta-analysis to determine the long-term efficacy and safety of colchicine for the secondary prevention of vascular disease.

**Methods:**

Randomized controlled trials comparing the incidence of cardiovascular events between patients with clinically manifest vascular disease randomized to colchicine vs. placebo and ≥12-month follow-up were included. The primary efficacy endpoint is major adverse cardiovascular events (MACE) and includes cardiovascular mortality, MI, ischaemic stroke, and urgent coronary revascularization. The DerSimonian and Laird random effects model was used to calculate pooled effect estimates.

**Results:**

Six RCTs, with a pooled sample size of 21 800 patients, were included (colchicine *n* = 10 871; placebo *n* = 10 929). Over a follow-up of 12–34 months, colchicine reduced the incidence of MACE compared with placebo [pooled hazard ratio .75, 95% confidence interval (CI) .56–.93]. The reduction in cardiovascular events among colchicine patients was driven by reductions in MIs, ischaemic strokes, and urgent coronary revascularizations (*P* < .05 for all). No differences were detected for safety outcomes (*P* > .05 for all), including non-cardiovascular deaths (risk ratio 1.08, 95% CI .76–1.54).

**Conclusions:**

This updated meta-analysis of RCTs demonstrated a substantial reduction in MACE, MI, ischaemic stroke, and recurrent coronary revascularization with colchicine compared with placebo. Therefore, the results support the use of colchicine to reduce recurrent cardiovascular events.

## Structured Graphical Abstract

**structured graphical abstract:**
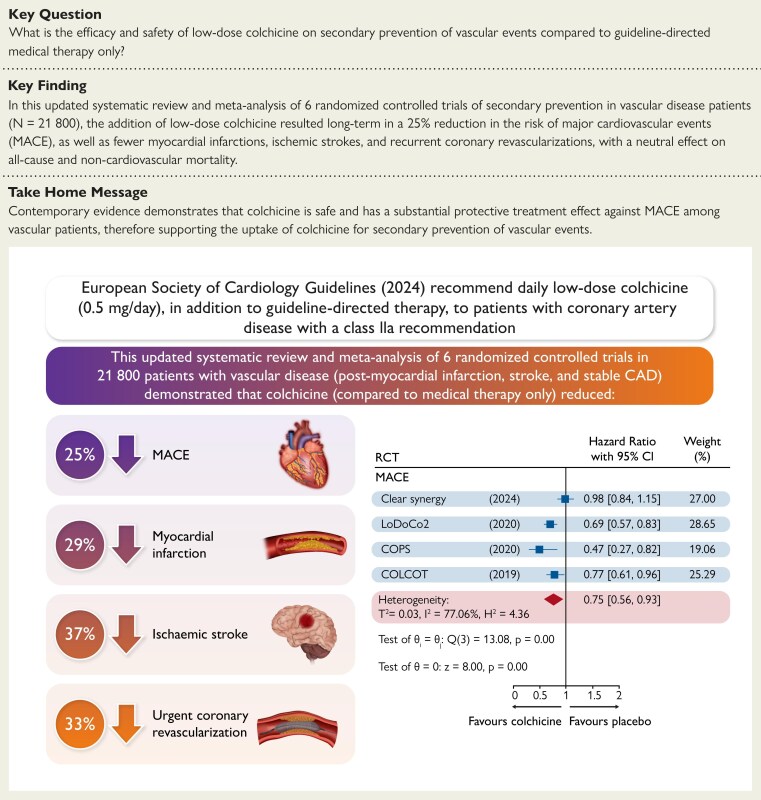

**See the editorial comment for this article ‘Colchicine in secondary prevention: making sense out of diversity’, by S.J. Pocock and G. Mendieta, https://doi.org/10.1093/eurheartj/ehaf266.**

## Introduction

Over the last decade, inflammation has emerged as an important therapeutic target for the management of atherosclerotic coronary artery disease (CAD).^1–3^ In a contemporary analysis of statin-treated atherosclerosis patients enrolled in large, randomized trials (*N* = 31 245), high-sensitivity C-reactive protein (hsCRP) as an inflammatory biomarker was shown to be a more powerful determinant of recurrent cardiovascular events, cardiovascular mortality, and all-cause mortality, than LDL-cholesterol.^4^ The efficacy of an intervention targeting inflammation in the CAD population was first demonstrated in the Canakinumab Anti-inflammatory Thrombosis Outcome Study (CANTOS; *N* = 10 061), which showed that inhibition of interleukin-1β with the monoclonal antibody canakinumab led to a 15% reduction in cardiovascular events among patients with hsCRP >2 mg/L.^5^ As such, the results of CANTOS were a proof-of-concept for the reduction of inflammation in secondary prevention of CAD and led to an increased focus on anti-inflammatory therapies to reduce the residual risk of cardiovascular events due to plaque destabilization and rupture.^1,3^

Colchicine has emerged as an inexpensive alternative anti-inflammatory treatment for CAD.^6–12^ Colchicine has an established safety profile and is indicated for the treatment of gout, familial Mediterranean fever, and pericarditis.^6–12^ The anti-inflammatory mechanism of colchicine is mediated by the inhibition of neutrophils and the release of inflammatory cytokines (interleukin-1 and 6).^1–3^ Several trials in the CAD population have shown that colchicine is effective in reducing major adverse cardiovascular events [MACE: cardiovascular death, stroke, myocardial infarction (MI), and revascularization], including in the post-MI and stable CAD populations.^6–9^ In a meta-analysis performed in 2021, low-dose colchicine (.5 mg daily) compared with placebo reduced MACE by 32%.^13^ As such, the European Society of Cardiology has included colchicine as a Class IIa recommendation for CAD patients.^14^ In addition, the US Food and Drug Administration has also approved colchicine for CAD patients. Despite guideline recommendations and available evidence demonstrating that colchicine provides additional protection against MACE, the uptake of colchicine in daily clinical practice remains limited.

The recently published Colchicine and Spironolactone in patients with Myocardial Infarction/Synergy Stent Registry (CLEAR-SYNERGY; *N* = 7062) randomized trial has provided further evidence for the use of colchicine in daily practice. CLEAR-SYNERGY investigated colchicine in the acute MI population.^15^ The trial was conducted during the COVID-19 pandemic and failed to detect the benefits of colchicine, providing inconsistent evidence about its efficacy profile. Therefore, the objective of the present study was to synthesize new evidence in a systematic review and meta-analysis to re-evaluate the efficacy and safety of colchicine for the secondary prevention of atherosclerotic vascular disease.

## Methods

### Search strategy

The meta-analysis was conducted in accordance with the Preferred Reporting Items for Systematic Reviews and Meta-Analyses (PRISMA) guidelines (checklist in Supplementary data online, *Table S1*). Randomized controlled trials (RCTs) comparing colchicine to placebo or no colchicine for secondary cardiovascular prevention were searched and selected in Medline (PubMed), EMBASE, and Cochrane Central (from inception to 5 November 2024). Citation chasing was conducted in Google Scholar, Scopus, and Web of Science to maximize the sensitivity of the search. Secondary prevention was defined as patients with clinically manifest or established vascular disease, including MI, stable CAD, and stroke. Query terms included ‘colchicine’, ‘coronary artery disease’, ‘acute coronary syndrome’, ‘myocardial infarction’, ‘cardiovascular disease’, ‘atherosclerosis’, ‘stroke’, and ‘secondary prevention’, either separately or in combination (detailed list provided in Supplementary data online, *Methods*).

### Study selection, data extraction, and bias assessment

Eligible RCTs included in the present meta-analysis: (i) compared daily use of primarily low-dose colchicine (.5 mg daily) to placebo or no colchicine for secondary cardiovascular prevention; (ii) reported at least one of the following outcomes: cardiovascular death, MI, stroke, cardiac arrest, or urgent coronary revascularization; (iii) treated all patients with guideline-directed medical therapy in addition to the randomized treatment; (iv) had a minimum follow-up of at least 12 months; and (v) were published in a peer-reviewed scientific journal. Similar to statin therapy, the benefits of colchicine are time-dependent, with anti-inflammatory effects on atherosclerotic plaques likely requiring at least 12 months.^16^ Therefore, the present meta-analysis focused on the long-term effects of colchicine, and trials such as CHANCE-3 with a short follow-up period of 3 months, were not included. Review articles, editorials, meta-analyses, observational studies, and published abstracts were excluded.

Two independent reviewers screened eligible trials and included an initial screening of titles and abstracts, followed by full-text reviews. The reviewers were not blinded to the titles of the journals, authors, or affiliated institutions. The reviewers reached a consensus on the included studies.

Study characteristics, patient characteristics, and outcomes were extracted from the intent-to-treat population for each included study. Study quality was assessed with the Cochrane Collaboration risk-of-bias tool for randomized trials.^17^

### Endpoints

The primary efficacy endpoint was MACE which included a composite of cardiovascular death, MI, ischaemic stroke, and urgent coronary revascularization. Secondary efficacy endpoints consisted of the components of the primary endpoint. The efficacy of colchicine among subgroups (i.e. age, sex, and diabetes) was only investigated for the primary composite endpoint. Safety outcomes were limited to serious adverse events (SAEs) and included hospitalizations for gastrointestinal events, infections, and pneumonia, as well as diagnosis of cancer.

### Strategies for data synthesis, statistical analyses, and evaluation of evidence

Pooled hazard ratios (HRs) with corresponding 95% confidence intervals (CIs) were calculated for the primary and secondary efficacy endpoints with the DerSimonian and Laird random-effects model. Risk ratios (RRs) and 95% CIs were also computed with the DerSimonian and Laird random-effects model. Higgins *I*^2^ statistic was used to evaluate heterogeneity, with *I*^2^ values stratified as <25%, 25%–75%, and >75% for low, moderate, and high degrees of heterogeneity, respectively. Further, inconsistency and variability in the treatment effect between subgroups were evaluated with the *Q*_b_ statistic.^18^ The Egger’s regression test and funnel plots were conducted to assess publication and small study bias.

Multiple sensitivity analyses were conducted to (i) exclude the CONVINCE trial (only trial of post-stroke patients), (ii) include only pre-COVID-19 results, and (iii) include RCTs in patients with chronic coronary disease. In addition, RRs and 95% CIs were calculated for modified primary (i.e. cardiovascular mortality, MI, ischaemic stroke, and coronary revascularization) and secondary efficacy endpoints, as defined in a meta-analysis performed by Fiolet *et al*.^19^ Statistical analyses were conducted with Stata (Version 16, StataCorp, College Station, TX, USA).

## Results

### Literature search results and risk of bias

Of 298 studies identified, 6 RCTs met all the inclusion criteria and none of the exclusion criteria. The characteristics of included RCTs are listed in *Table 1*. All trials investigated colchicine in secondary prevention among populations with atherosclerotic disease, including post-MI [Colchicine Cardiovascular Outcomes Trial (COLCOT) and CLEAR-SYNERGY], post-stroke (CONVINCE), stable CAD [Low-Dose Colchicine (LoDoCo) and Low-Dose Colchicine 2 (LoDoCo2)], and acute coronary syndrome [COlchicine in Patients with acute coronary Syndrome (COPS)].^6–9,15,20^

**Table 1:** Study characteristics

	LoDoCo^6^	COLCOT^7^	COPS^8^	LoDoCo2^9^	CONVINCE^20^	CLEAR^15^
Year	2013	2019	2020	2020	2024	2025
Study design	Single-blind RCT	Double-blind, placebo controlled RCT	Double-blind, placebo controlled RCT	Double-blind, placebo controlled RCT	Double-blind, placebo controlled RCT	Double-blind, 2 × 2 factorial design, placebo controlled RCT
Study population	Stable CAD	Recent MI (<30 days)	ACS	Stable CAD	Post-stroke	Post-MI
*N*	532	4745	795	5522	3144	7062
Key inclusion criteria	Angiographically proven CAD; clinically stable for ≤6 months	MI within 30 days prior; completed planned percutaneous revascularization procedures; treated according to national guidelines that included intensive use of statins	ACS (STEMI/NSTEMI/UA); evidenced CAD on coronary angiography, managed with either PCI or medical therapy	Evidence of CAD on invasive angiography or computed tomography angiography or a coronary calcium score of ≥400 Agatston units on a coronary artery calcium scan; clinically stable condition for ≥6 months	Non-severe ischaemic stroke (modified Rankin Scale score ≤3) or high risk of transient ischaemic attack (ABCD2 score of ≥4); clinically stable; time between qualifying event and randomization of 72 h to 28 days	STEMI or NSTEMI and underwent a percutaneous coronary intervention; and 1 risk factor: left ventricular ejection fraction ≤45%, diabetes, multivessel coronary disease, prior MI, or age ≥60 years
Key exclusion criteria	Bypass surgery within 10 years prior	Severe heart failure; left ventricular ejection fraction <35%; stroke within the previous 3 months; Type 2 index MI; coronary bypass surgery either within previous 3 years or planned; severe renal or hepatic disease; known cancer	CAD requiring surgical revascularization pre-existing long-term colchicine use or immunosuppressant therapy; severe hepatic or renal insufficiency; known active malignancy	Moderate-to-severe renal impairment; severe heart failure; severe valvular disease	Stroke or TIA caused by atrial fibrillation, or cardiac embolism, etc.; pre-existing moderate-to-severe renal, liver, or blood disorders, inflammatory bowel disease or chronic diarrhoea	Active diarrhoea, contraindication to everolimus, unable to receive dual antiplatelet therapy, history of cirrhosis or current severe hepatic disease, creatinine clearance <30 mL/min/1.73 m^2^
Median follow-up	24 months	22.6 months	12 months	28.6 months	33.6 months	26 months
Primary endpoint	Composite: ACS, out-of-hospital cardiac arrest, or non-cardioembolic ischaemic stroke	Composite: CV mortality, resuscitated cardiac arrest, MI, stroke, or urgent hospitalization for angina requiring revascularization	Composite: all-cause mortality, ACS (STEMI/NSTEMI/UA), ischaemia-driven revascularization, or non-cardioembolic ischaemic stroke	Composite: CV death, MI, ischaemic stroke, or ischaemia-driven coronary revascularization	Composite: first fatal or non-fatal recurrent ischaemic stroke, MI, cardiac arrest, CV mortality, and hospitalization for unstable angina	Composite: CV mortality, MI, stroke, or ischaemia-driven revascularization

ACS, acute coronary syndrome; CAD, coronary artery disease; CV, cardiovascular; MI, myocardial infarction; NSTEMI, non-ST-elevation myocardial infarction; RCT, randomized controlled trial; STEMI, ST-elevation myocardial infarction; TIA transient ischaemic attack; UA, unstable angina.

The pooled sample size from the included trials was 21 800 patients. A total of 10 871 patients were randomized to colchicine, and 10 929 patients were randomized to placebo (or no colchicine, in the LoDoCo trial only). Patients were followed from 12 to 33.6 months, depending on the trial. The Cochrane Collaboration risk-of-bias assessment tool classified five trials as ‘low risk’ for overall bias^7–9^ and one trial as a potential for ‘some concern’^6^ (see Supplementary data online, *Table S2*).

### Patient characteristics

Baseline patient characteristics for each RCT are summarized in *Table 2*. The mean age of patients ranged from 60 to 67 years and more than 90% of patients were on concomitant statin therapy. The CONVINCE trial of prior stroke patients differed from the other included trials with the inclusion of more women (30% vs. <21%) and a higher prevalence of hypertension (65% vs. <52%; CONVINCE vs. other trials; *Table 2*). The CONVINCE trial did not report the history of percutaneous coronary intervention (PCI) and coronary artery bypass grafting (CABG), however, amongst the other included studies, the LoDoCo and LoDoCo2 trials for stable CAD patients had a substantially higher rate of prior PCI (>55% vs. <18%) and prior CABG (>11% vs. <5%) than the other trials, respectively (*Table 2*). The distribution of all other patient characteristics was relatively similar between trials.

**Table 2:** Patient characteristics at baseline

	LoDoCo^6^		COLCOT^7^		COPS^8^		LoDoCo2^9^		CONVINCE^20^		CLEAR^15^	
	Colchicine (*N* = 250)	Control (*N* = 282)	Colchicine (*N* = 2366)	Placebo (*N* = 2379)	Colchicine (*N* = 396)	Placebo (*N* = 399)	Colchicine (*N* = 2762)	Placebo (*N* = 2760)	Colchicine (*N* = 1569)	Placebo (*N* = 1575)	Colchicine (*N* = 3528)	Placebo (*N* = 3534)
Age, years (mean ± SD)	67 ± 9.2	66 ± 9.6	60.6 ± 10.7	60.5 ± 10.6	59.7 ± 10.2	60.0 ± 10.4	65.8 ± 8.4	65.9 ± 8.7	66.4 ± 10.0	66.2 ± 9.9	60.6 ± 10.3	60.7 ± 10.3
Women (%)	11.2	11.0	19.9	18.4	18.7	22.3	16.5	14.1	31.1	29.5	20.5	20.2
Hypertension (%)	Not reported	Not reported	50.1	52.0	50.8	49.9	51.4	50.3	65.4	65.5	45.9	45.6
Diabetes (%)	27.6	32.6	19.5	20.9	18.9	19.0	17.8	18.7	22.8	21.8	18.7	18.3
History of PCI (%)	55.2	59.9	16.6	17.1	12.9	12.5	76.0	75.3	Not reported	Not reported	9.8	9.2
History of CABG (%)	15.6	22.0	2.9	3.4	3.8	4.8	11.5	14.2	Not reported	Not reported	Not reported	Not reported
Statin use (%)	94.0^a^	96.1^a^	98.9	99.1	98.2	99.5	93.9	94.0	93.7	94.0	96.6	96.7

CABG, coronary artery bypass graft; PCI, percutaneous coronary intervention; SD, standard deviation.

^a^The LoDoCo trial only reported the use of high-dose statins, not any statin use.

### Clinical efficacy endpoints

**Figure 1:**
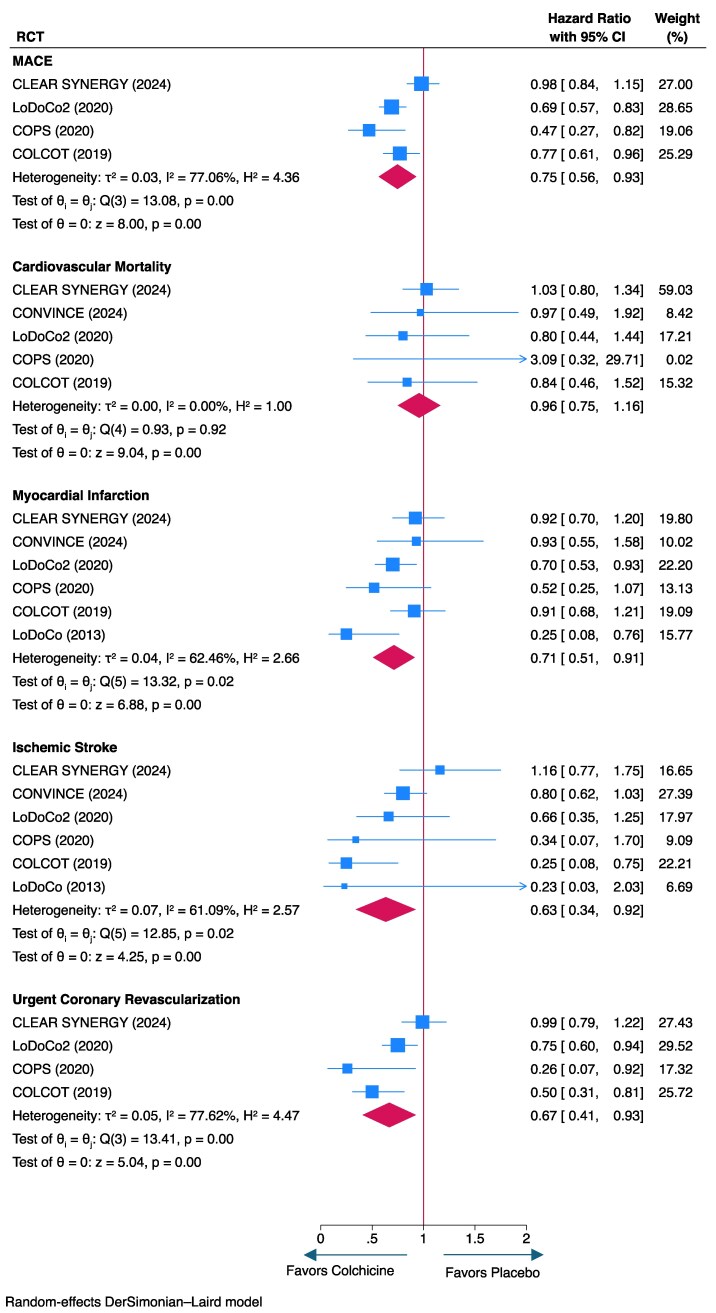
Forest plot of clinical efficacy endpoints (hazard ratios)

For the primary efficacy endpoint of MACE (i.e. composite of cardiovascular death, MI, ischaemic stroke, and urgent coronary revascularization), colchicine was associated with a significant reduction of 25% compared with patients assigned to placebo or no colchicine (pooled HR .75, 95% CI .56–.93; *I*^2^ = 77.1%; *Figure 1*). In addition, colchicine was associated with significant reductions in the incidence of MIs (pooled HR, .71; 95% CI, .51–.91; *I*^2^ = 62.5%), ischaemic strokes (pooled HR, .63; 95% CI, .34–.92; *I*^2^ = 61.1%), and urgent coronary revascularizations (pooled HR, .67; 95% CI, .41–.93; *I*^2^ = 77.6%; *Figure 1*). However, no significant difference was detected for cardiovascular mortality (*Figure 1*).

**Figure 2:**
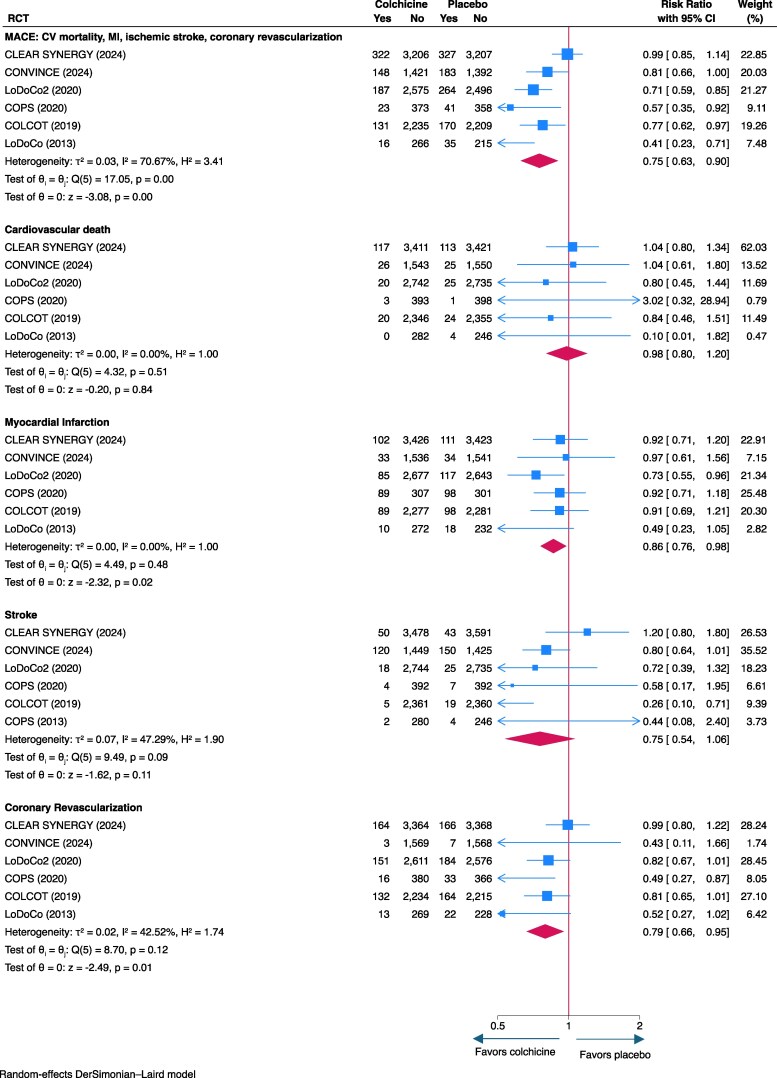
Forest plot of clinical efficacy endpoints (risk ratios)

The results for MACE (i.e. composite of MI, ischaemic stroke, coronary revascularization, and cardiovascular death), MI, and coronary revascularization were consistent in the sensitivity analysis for pooled risk ratios that included all trials; however, the protective effect against stroke did not reach statistical significance (*Figure 2*). In a sensitivity analysis excluding the post-stroke CONVINCE trial, the magnitude of the protective effect of colchicine increased, and heterogeneity (*I*^2^) between studies decreased (see Supplementary data online, *Figure S1*). In addition, pooled pre-COVID results including those from CLEAR-SYNERGY (*n* = 1989 of 7062 patients included) demonstrated a greater reduction in MACE with colchicine compared with placebo (HR, .70; 95% CI, .60–.81; Supplementary data online, *Figure S2*). In addition, heterogeneity between pooled studies was substantially lower in the pre-COVID sensitivity analysis (*I*^2^ = 22.59%) compared with the main analysis that included all patients (*I*^2^ = 77.06%). There was a trend towards a statistically significant difference detected in trials that only included acute coronary disease patients only (HR, .76; 95% CI, .50–1.02; Supplementary data online, *Figure S3*).

Visual inspection of funnel plots showed they were symmetrical, and the results of the Egger’s test indicated there was no significant risk of publication bias for all outcomes (*P* > .05 for all; Supplementary data online, *Figure S4*).

### Subgroup analyses

**Figure 3:**
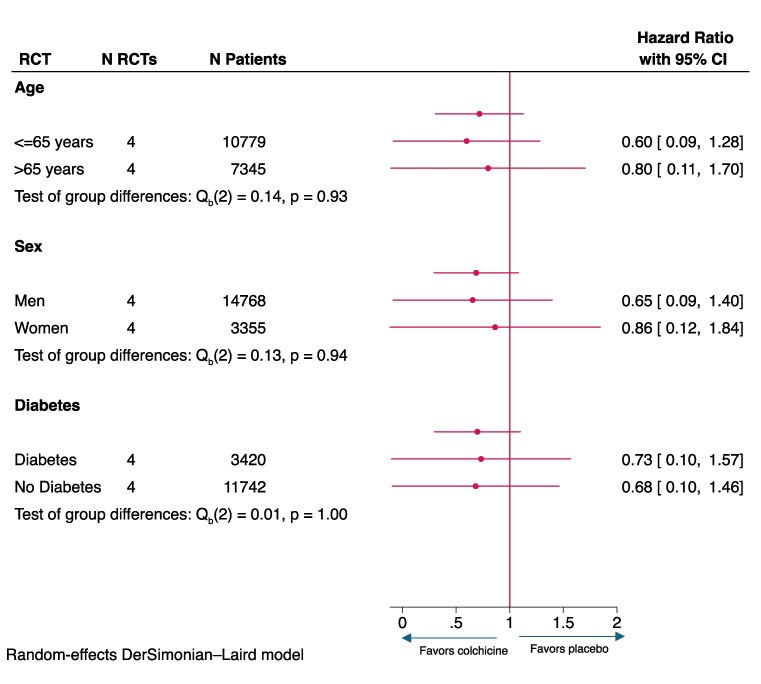
Forest plot of subgroup analyses (primary composite outcome)

The was no significant difference in the incidence of the primary composite cardiovascular endpoint for age, sex, and diabetes strata (*Figure 3*). All analyses were not powered for subgroup analyses, and further, too few women enrolled in trials to evaluate an effect (*Figure 3*). Overall, the *Q*_b_ statistic suggests that there is minimal variability and inconsistency in the treatment effect between strata in subgroup analyses (*P* > .05 for all).

### Major adverse events

**Figure 4:**
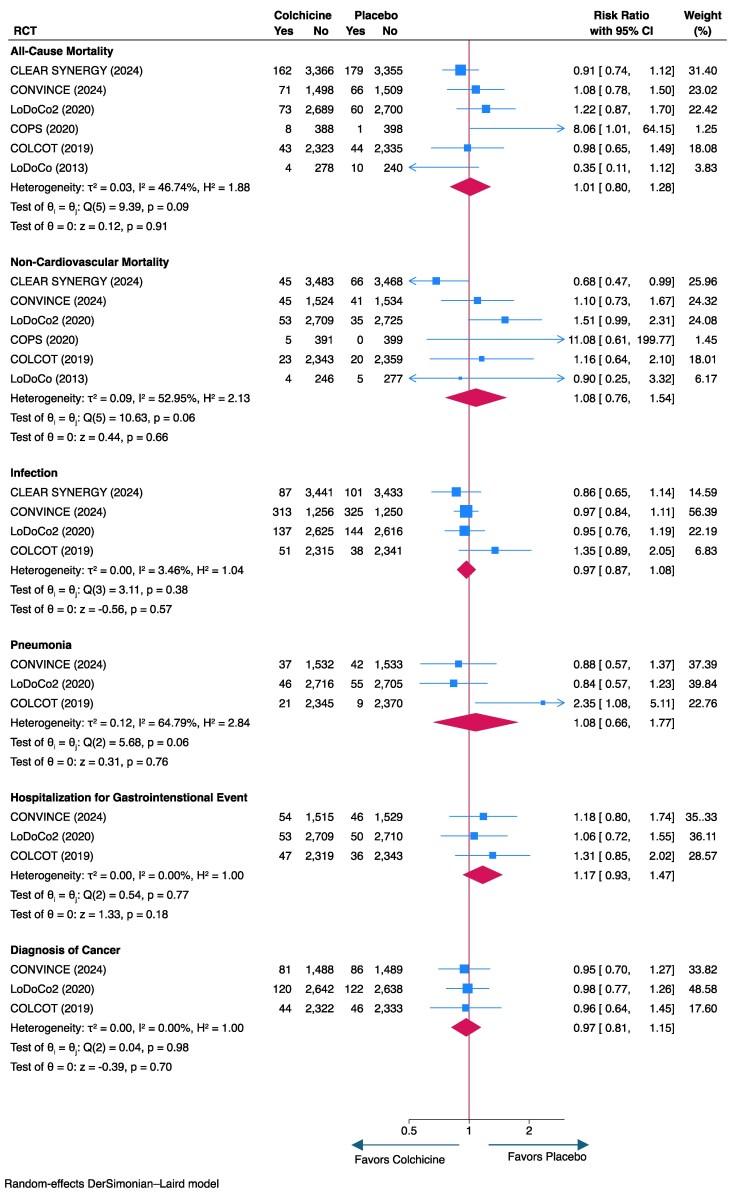
Forest plot of serious adverse events

No significant differences were detected for all-cause mortality (RR, 1.01; 95% CI, .80–1.28), non-cardiovascular mortality (RR, 1.08; 95% CI, .76–1.54), infections, pneumonias, hospitalizations for gastrointestinal events, and diagnoses of cancer between patients randomized to colchicine or placebo (*Figure 4*). Moderate degrees of heterogeneity were detected for all-cause (*I*^2^ = 46.7%) and non-cardiovascular (*I*^2^ = 53.0%) mortality as well as pneumonia (*I*^2^ = 64.8%) (*Figure 4*).

## Discussion

In this updated meta-analysis of RCTs comparing colchicine to placebo (or no colchicine) for secondary cardiovascular prevention, we demonstrated that: (i) treatment with colchicine reduced the incidence of MACE by 25%; (ii) colchicine was primarily associated with fewer MIs, ischaemic strokes, and urgent coronary revascularizations; (iii) no heterogeneity in treatment effect was detected by age, sex, or diabetes, and (iv) colchicine was relatively safe, including no difference in all-cause and non-cardiovascular deaths (*Structured Graphical Abstract*). Although the addition of newer trials attenuated the magnitude of the pooled protective effect of colchicine, reductions in cardiovascular events remained substantial, further supporting the use of colchicine in combination with guideline-directed medical therapy to decrease cardiovascular events in the atherosclerotic vascular disease population.

### Colchicine in patients with coronary artery disease

This updated meta-analysis (including CLEAR-SYNERGY) shows the large benefits of low-dose colchicine on cardiovascular events in patients with CAD. The neutral results of CLEAR-SYNERGY^15^ are discordant with those of COLCOT, LoDoCo2 and a recent meta-analysis that included 14 738 patients and 1198 cardiovascular events.^7,9,19^ In CLEAR-SYNERGY, there was a reduction of 22% in the incidence of a primary endpoint event with colchicine vs. placebo before the COVID-19 pandemic, which was lost during the pandemic, with an interaction between the COVID-19 phase and the treatment effect (*P* < .10). The inverted relationship between the incidence of non-fatal MI and all-cause deaths in CLEAR-SYNERGY is consistent with the published impact of COVID-19 on cardiovascular clinical care and under-reporting of non-fatal events.^21,22^ The same phenomenon related to the pandemic was observed in the Dal-GenE-1 study, in which the MI/all-death ratio decreased from 1.74 before the COVID-19 pandemic to .96 during the pandemic.^23^ In CLEAR-SYNERGY, the MI/all-cause death ratio was even more markedly inverted at .62, with rates of MI and death at 3.1% and 5.1% for a median follow-up of 3 years. In contrast, the COMPLETE trial conducted before the COVID era reported rates of MI and death of 7.9% and 5.2% with the same median follow-up of 3 years.^24^ These data indicate major under-reporting of MI during the pandemic in CLEAR-SYNERGY. Dal-GenE-1, IRONMAN, and GUIDE-HF are other published trial results that were also affected by the COVID-19 pandemic, with a loss of significance during the pandemic.^23,25,26^

Furthermore, inflammation was not controlled in the colchicine arm of CLEAR-SYNERGY with a least-squares mean hsCRP of 3.0 mg/L (95% CI 2.6–3.5), in contrast to the on-treatment median values of 1.12 mg/L [interquartile range (IQR): .77–2.10] in COLCOT and .94 mg/L (IQR: .53–1.93) in LoDoCo2. These data are important, given that CANTOS has previously shown that inflammation reduction therapy lowers the incidence of cardiovascular events only in those with an on-treatment hsCRP <2.0 mg/L (as in COLCOT and LoDoCo2), but not in those with hsCRP ≥ 2.0 mg/L (as in CLEAR-SYNERGY).^27^ The effect of COVID-19 and the inadequate reduction of inflammation in CLEAR-SYNERGY make its results uninterpretable. As such, whether the results of CLEAR-SYNERGY should have been included in a meta-analysis is uncertain. Nevertheless, the inclusion of these data in the current meta-analysis still yielded a significant reduction of 25% in the primary efficacy endpoint with colchicine compared with placebo.

### Colchicine in the stroke population

CONVINCE was the only trial that investigated with at least 1 year of follow-up the effects of colchicine in hospital-based patients with non-severe cardioembolic stroke or high-risk transient ischaemic attack, a population that constituted only 2%–3% of colchicine trials for the secondary prevention of CAD.^20^ In COLCOT, colchicine reduced vascular recurrent events, including ischaemic strokes (HR, .26; 95% CI, .10–.70).^7^ Although atherosclerosis is not the only cause of stroke, inflammation was shown to have a role in small vessel disease or cryptogenic stroke, therefore it is plausible that a similar mechanism of inflammation inhibition may decrease recurrent strokes.

The prior stroke population in the CONVINCE trial differed from colchicine CAD trials, with a greater proportion of women and a higher prevalence of hypertension.^20^ Further, CONVINCE did not include haemorrhagic strokes, like the COLCOT and CLEAR-SYNERGY trials.^7,15,20^ A trend towards a reduction in a composite of recurrent non-fatal ischaemic stroke, MI, cardiac arrest, vascular death or hospitalization for unstable angina was detected among patients randomized to colchicine compared with placebo (HR, .84; 95% CI, .68–1.05).^20^ The trend seemed to be driven by all ischaemic strokes and non-fatal ischaemic strokes that also trended towards a reduction with colchicine (HR, .80; 95% CI, .68–1.05); however, CONVINCE was not powered to detect an effect on the component outcome of stroke.^20^ A statistically significant reduction of the primary composite endpoint was detected in the on-treatment analysis of the CONVINCE trial (aHR, .80; 95% CI, .53–.99); however, on-treatment results for stroke and non-fatal ischaemic strokes were not presented.^20^ Therefore, the results of the CONVINCE trial suggest that further trials of colchicine are warranted among patients with minor atherosclerotic stroke.

In addition, CONVINCE patients treated with colchicine had a greater decrease in hsCRP compared with guideline-directed therapy only, a reduction captured at 1-month post-baseline and persisted throughout 3 years of follow-up.^20^ Although the overall results for colchicine in the prior stroke population are inconclusive with the CONVINCE trial, indication for a protective effect with colchicine warrants additional trials to further elucidate the effect.

### Treatment effects across subgroups

Despite increased power to detect heterogeneity in treatment effects by age, sex, and diabetes, efficacy did not differ across subgroups. There was still no significant difference detected for colchicine in women; however, there was limited statistical power as women constituted only 20% of the population enrolled in the trials. As a result, with no significant heterogeneity between subgroups, widespread use of colchicine for secondary prevention may be considered.

### Safety

Colchicine has been used for centuries to treat gout and has an established safety profile. Only the COLCOT trial detected a difference in the rate of pneumonia between patients randomized to colchicine (.9%) or placebo (.4%); however, unlike CANTOS, there were no fatal infections and further, the low event rate may have resulted in statistical significance due to chance.^5,7^ Updated pooled analyses did not show an increased risk of any serious adverse event; however, there was a trend towards an increase in hospitalization for gastrointestinal events that did not reach statistical significance. There was no evidence of an increased risk of gastrointestinal cancer or other gastrointestinal disorders (e.g. diverticular disease or biliary disease). As a similar trend of increased rate of hospitalizations for gastrointestinal disease has not been observed in studies of the long-term treatment of familial Mediterranean fever, it is plausible that the result is due to chance from a low incidence of events or to the prevalence in CLEAR-SYNERGY of COVID-19 which often presents with gastrointestinal symptoms.^1,2^ Importantly, there was no effect on all-cause and non-cardiovascular mortality. Overall, the pooled results from the present meta-analysis further demonstrates that colchicine is safe to use in patients with atherosclerotic cardiovascular disease and can be initiated soon after MI, as evidenced in the CLEAR-SYNERGY trial. Early intestinal intolerance of colchicine is typically mild and self-limiting.

### Limitations

While the present meta-analysis provides the most contemporary, comprehensive estimates of the efficacy and safety of colchicine among patients with atherosclerotic vascular disease, key limitations need to be considered. The Higgins *I*^2^ statistics showed that pooled estimates for many efficacy endpoints had a moderate degree of heterogeneity. There are multiple factors that may have influenced heterogeneity including differences in the target populations, variations in dosing or discontinuation rates, degree of inflammation as measured by C-reactive protein, the presence of newer inflammatory diseases such as COVID-19, and the effect of an unexpected pandemic on the reporting of cardiovascular events. Although the included trials investigated target populations with similar mechanisms of action in vascular disease, we performed multiple sensitivity analyses to reduce heterogeneity, including the exclusion of CONVINCE (i.e. stroke population) and pre-COVID-19 effect estimates, which showed that the results were consistent. It should be noted that assessments of heterogeneity may also not be optimal due to the number of studies included in the meta-analysis.

Further, strict endpoint definitions were used to ensure consistency and accuracy of effect estimates. Nevertheless, this led to the exclusion of key trials from primary efficacy analyses, particularly the CONVINCE trial. Therefore, to determine the impact of the CONVINCE trial on the efficacy endpoints, sensitivity analyses using risk ratios, rather than hazard ratios, were calculated based on new endpoint definitions from a meta-analysis performed by Fiolet *et al*.^19^ Also, a few subgroups could be investigated due to limited consistent subgroup analyses in the CONVINCE and CLEAR-SYNERGY trials for the primary endpoint of MACE. Finally, due to the few eligible trials, a meta-regression analysis to assess effect modification could not be performed; however, potential effect modification was not detected in subgroup analyses.

## Conclusions

The inclusion of results from new large, randomized trials provides further robust evidence that the addition of low-dose colchicine to standard-of-care therapy decreases the incidence of MACE, MI, ischaemic stroke, and recurrent coronary revascularization in vascular disease patients. Compared with pooled results from prior trials, the inclusion of these additional studies attenuated slightly the overall magnitude of the effect of colchicine, with a reduction in MACE of 25%; however, the pooled efficacy of colchicine in vascular disease patients remains substantial, with a greater protective effect than intensive lipid-lowering therapies, such as proprotein convertase subtilisin/kexin type 9 inhibitors. In addition, this pooled analysis did not show an excess in all-cause and non-cardiovascular mortality. Although the recently reported CLEAR-SYNERGY trial by itself did not show an effect, the higher level of evidence from pooled results in a meta-analysis of RCTs indicates that long-term treatment with colchicine provides robust benefits to vascular disease patients, as described in current clinical guideline recommendations. As such, contemporary safety and efficacy evidence supports the uptake of colchicine for the secondary prevention of vascular disease.

## Supplementary Material

ehaf174_Supplementary_Data

## Declarations

### Data Availability

The data underlying this article are available in the article and in its online Supplementary material.
