# Supplementary material for: Long-term trials of colchicine for secondary prevention of vascular events: a meta-analysis
Source: Eur Heart J. 2025 May 2;46(26):2552–63. doi: 10.1093/eurheartj/ehaf174 (PMC12233006; doi:10.1093/eurheartj/ehaf174)
Supplement: ehaf174_Supplementary_Data [file ehaf174_supplementary_data.docx]

**SUPPLEMENTAL MATERIAL**

Search strategies …………………………………………………………………………………………2

PRISMA checklist………………………………………………………………………………………. 3

Cochrane collaboration risk-of-bias assessment………………………………………………………... 7

Sensitivity analysis for efficacy outcomes:

**Excludes** CONVINCE trial…………………………………….................................................19

Pre-COVID results ..……………………………………………................................................20

Bias assessments- Funnel plot and Eggers Test……………………………………………………..… 21

**Supplementary Methods:**

**Search Strategy**

Search terms included:

| “colchicine” [all fields] OR “colchicine” [MeSH terms] |
| --- |
| “coronary artery disease” [all fields] OR “coronary artery disease” [MeSH terms] OR “CAD” [all fields] or “Percutaneous coronary intervention” [all fields] OR “Percutaneous coronary intervention” [MeSH] or “PCI” [all fields] OR “acute coronary syndrome” [MeSH terms] OR “acute coronary syndrome” [all fields] or “ACS” [all fields] or “myocardial infarction” [MeSH terms] or “MI” [all fields] |
| “clinical trial” [MeSH] or “randomized controlled trial” [MeSH] or “randomized controlled trial” [all fields] |
| “human” [MeSH] NOT “animal” [MeSH] |


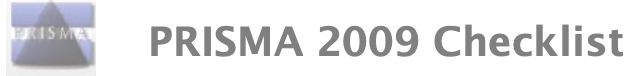
**Supplemental Table S1.** PRISMA Checklist

| **Section/topic** | **#** | **Checklist item** | **Reported on page #** |
| --- | --- | --- | --- |
| **TITLE** | | |  |
| Title | 1 | Identify the report as a systematic review, meta-analysis, or both. | 1 |
| **ABSTRACT** | | |  |
| Structured summary | 2 | Provide a structured summary including, as applicable: background; objectives; data sources; study eligibility criteria, participants, and interventions; study appraisal and synthesis methods; results; limitations; conclusions and implications of key findings; systematic review registration number. | 3 |
| **INTRODUCTION** | | |  |
| Rationale | 3 | Describe the rationale for the review in the context of what is already known. | 6-7 |
| Objectives | 4 | Provide an explicit statement of questions being addressed with reference to participants, interventions, comparisons, outcomes, and study design (PICOS). | 7 |
| **METHODS** | | |  |
| Protocol and registration | 5 | Indicate if a review protocol exists, if and where it can be accessed (e.g., Web address), and, if available, provide registration information including registration number. | ND |
| Eligibility criteria | 6 | Specify study characteristics (e.g., PICOS, length of follow-up) and report characteristics (e.g., years considered, language, publication status) used as criteria for eligibility, giving rationale. | 7 |
| Information sources | 7 | Describe all information sources (e.g., databases with dates of coverage, contact with study authors to identify additional studies) in the search and date last searched. | 7 |
| Search | 8 | Present full electronic search strategy for at least one database, including any limits used, such that it could be repeated. | 7 & supplement |
| Study selection | 9 | State the process for selecting studies (i.e., screening, eligibility, included in systematic review, and, if applicable, included in the meta-analysis). | 7 |
| Data collection process | 10 | Describe method of data extraction from reports (e.g., piloted forms, independently, in duplicate) and any processes for obtaining and confirming data from investigators. | 7 |
| Data items | 11 | List and define all variables for which data were sought (e.g., PICOS, funding sources) and any assumptions and simplifications made. | 7-8 |
| Risk of bias in individual studies | 12 | Describe methods used for assessing risk of bias of individual studies (including specification of whether this was done at the study or outcome level), and how this information is to be used in any data synthesis. | 8 |
| Summary measures | 13 | State the principal summary measures (e.g., risk ratio, difference in means). | 8 |
| Synthesis of results | 14 | Describe the methods of handling data and combining results of studies, if done, including measures of consistency (e.g., I^2^) for each meta-analysis. | 8 |

| **Section/topic** | **#** | **Checklist item** | **Reported on page #** |
| --- | --- | --- | --- |
| **TITLE** | | |  |
| Title | 1 | Identify the report as a systematic review, meta-analysis, or both. | 1 |
| **ABSTRACT** | | |  |
| Structured summary | 2 | Provide a structured summary including, as applicable: background; objectives; data sources; study eligibility criteria, participants, and interventions; study appraisal and synthesis methods; results; limitations; conclusions and implications of key findings; systematic review registration number. | 3 |
| **INTRODUCTION** | | |  |
| Rationale | 3 | Describe the rationale for the review in the context of what is already known. | 6-7 |
| Objectives | 4 | Provide an explicit statement of questions being addressed with reference to participants, interventions, comparisons, outcomes, and study design (PICOS). | 7 |
| **METHODS** | | |  |
| Protocol and registration | 5 | Indicate if a review protocol exists, if and where it can be accessed (e.g., Web address), and, if available, provide registration information including registration number. | ND |
| Eligibility criteria | 6 | Specify study characteristics (e.g., PICOS, length of follow-up) and report characteristics (e.g., years considered, language, publication status) used as criteria for eligibility, giving rationale. | 7 |
| Information sources | 7 | Describe all information sources (e.g., databases with dates of coverage, contact with study authors to identify additional studies) in the search and date last searched. | 7 |
| Search | 8 | Present full electronic search strategy for at least one database, including any limits used, such that it could be repeated. | 7 & supplement |
| Study selection | 9 | State the process for selecting studies (i.e., screening, eligibility, included in systematic review, and, if applicable, included in the meta-analysis). | 7 |
| Data collection process | 10 | Describe method of data extraction from reports (e.g., piloted forms, independently, in duplicate) and any processes for obtaining and confirming data from investigators. | 7 |
| Data items | 11 | List and define all variables for which data were sought (e.g., PICOS, funding sources) and any assumptions and simplifications made. | 7-8 |
| Risk of bias in individual studies | 12 | Describe methods used for assessing risk of bias of individual studies (including specification of whether this was done at the study or outcome level), and how this information is to be used in any data synthesis. | 8 |
| Summary measures | 13 | State the principal summary measures (e.g., risk ratio, difference in means). | 8 |
| Synthesis of results | 14 | Describe the methods of handling data and combining results of studies, if done, including measures of consistency (e.g., I^2^) for each meta-analysis. | 8 |


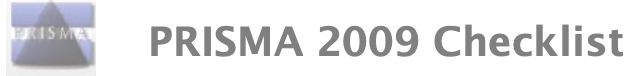


*From:*  Moher D, Liberati A, Tetzlaff J, Altman DG, The PRISMA Group (2009). Preferred Reporting Items for Systematic Reviews and Meta-Analyses: The PRISMA Statement. PLoS Med 6(7): e1000097. doi:10.1371/journal.pmed1000097

For more information, visit: **www.prisma-statement.org**.

**Supplemental Table S2.** Cochrane Collaboration Risk-of-Bias Tool

| **Study details**   \| **Reference** \| Ref 16- Jolly SS et al. *N Engl J Med* 2024  Ref 15- Kelly P et al. *Lancet* 2024  Ref 10 – Tardif JC et al. *N Engl J Med* 2019  Ref 12 – Nidorf SM et al. *N Engl J Med* 2020  Ref 11 – Nidorf SM et al. *J Am Coll Cardiol* 2013  Ref 13 – Tong DC et al. *Circulation*. 2020 \| \| --- \| --- \|   **Study design**   \| ⮽ \| Individually-randomized parallel-group trial \| \| --- \| --- \| \| □ \| Cluster-randomized parallel-group trial \| \| □ \| Individually randomized cross-over (or other matched) trial \|  \| **Specify which outcome is being assessed for risk of bias** \| Composite: Cardiovascular mortality, myocardial infarction, ischemic stroke, urgent coronary revascularization \| \| --- \| --- \|  \| **Specify the numerical result being assessed.** In case of multiple alternative analyses being presented, specify the numeric result (e.g. RR = 1.52 (95% CI 0.83 to 2.77) and/or a reference (e.g. to a table, figure or paragraph) that uniquely defines the result being assessed. \| HR, 0.75; 95% CI, 0.56-0.93; I^2^=77.06% \| \| --- \| --- \|   **Is the review team’s aim for this result…?**   \| ⮽ \| to assess the effect of *assignment to intervention* (the ‘intention-to-treat’ effect) \| \| --- \| --- \| \| □ \| to assess the effect of *adhering to intervention* (the ‘per-protocol’ effect) \|   **Which of the following sources were obtained to help inform the risk-of-bias assessment? (tick as many as apply)**  ⮽ Journal article(s) with results of the trial  ⮽ Trial protocol  ⮽ Statistical analysis plan (SAP)  □ Non-commercial trial registry record (e.g. ClinicalTrials.gov record)  □ Company-owned trial registry record (e.g. GSK Clinical Study Register record)  □ “Grey literature” (e.g. unpublished thesis)  □ Conference abstract(s) about the trial  □ Regulatory document (e.g. Clinical Study Report, Drug Approval Package)  □ Research ethics application  □ Grant database summary (e.g. NIH RePORTER or Research Councils UK Gateway to Research)  □ Personal communication with trialist  □ Personal communication with the sponsor |
| --- | --- | --- | --- | --- | --- | --- | --- | --- | --- | --- | --- | --- | --- | --- | --- | --- |

**Domain 1: Risk of bias arising from the randomization process**

| **Signaling questions** | **LoDoCo** | **COLCOT** | **COPS** | **LoDoCo2** | **CONVINCE** | **CLEAR SYNERGY** |
| --- | --- | --- | --- | --- | --- | --- |
| **1.1 Was the allocation sequence random?** | Y | Y | Y | Y | Y | Y |
| **1.2 Was the allocation sequence concealed until participants were enrolled and assigned to interventions?** | Y | Y | Y | Y | Y | Y |
| **1.3 Did baseline differences between intervention groups suggest a problem with the randomization process?** | N | N | N | N | N | N |
| **Risk-of-bias judgement** | Low | Low | Low | Low | Low | Low |
| Optional: What is the predicted direction of bias arising from the randomization process? | Favors experimental / Favors comparator / Toward null /Away from null / Unpredictable | Favors experimental / Favors comparator / Toward null /Away from null / Unpredictable | Favors experimental / Favors comparator / Toward null /Away from null / Unpredictable | Favors experimental / Favors comparator / Toward null /Away from null / Unpredictable | Favors experimental / Favors comparator / Toward null / Away from null / Unpredictable | Favors experimental / Favors comparator / Toward null / Away from null / Unpredictable |

Domain 2: Risk of bias due to deviations from the intended interventions (*effect of assignment to intervention*)

| **Signaling questions** | **LoDoCo** | **COLCOT** | **COPS** | **LoDoCo2** | **CONVINCE** | **CLEAR SYNERGY** |
| --- | --- | --- | --- | --- | --- | --- |
| **2.1. Were participants aware of their assigned intervention during the trial?** | Y | N | N | N | N | N |
| **2.2. Were carers and people delivering the interventions aware of participants' assigned intervention during the trial?** | Y | N | N | N | N | N |
| **2.3. If Y/PY/NI to 2.1 or 2.2: Were there deviations from the intended intervention that arose because of the experimental context?** | Y | Y | Y | Y | Y | Y |
| **2.4. If Y/PY to 2.3: Were these deviations from intended intervention balanced between groups?** | PN | PN | PN | PN | PN | PN |
| **2.5 If N/PN/NI to 2.4: Were these deviations likely to have affected the outcome?** | PN | PN | PN | PN | PN | PN |
| **2.6 Was an appropriate analysis used to estimate the effect of assignment to intervention?** | Y | Y | Y | Y | Y | Y |
| **2.7 If N/PN/NI to 2.6: Was there potential for a substantial impact (on the result) of the failure to analyze participants in the group to which they were randomized?** |  |  |  |  |  |  |
| **Risk-of-bias judgement** | Some concern | Low | Low | Low | Low | Low |
| Optional: What is the predicted direction of bias due to deviations from intended interventions? | Favors experimental / Favors comparator / Toward null /Away from null / Unpredictable | Favors experimental / Favors comparator / Toward null /Away from null / Unpredictable | Favors experimental / Favors comparator / Toward null /Away from null / Unpredictable | Favors experimental / Favors comparator / Toward null /Away from null / Unpredictable | Favors experimental / Favors comparator / Toward null / Away from null / Unpredictable | Favors experimental / Favors comparator / Toward null / Away from null / Unpredictable |

Domain 2: Risk of bias due to deviations from the intended interventions (*effect of adhering to intervention*)

| **Signaling questions** | **LoDoCo** | **COLCOT** | **COPS** | **LoDoCo2** | **CONVINCE** | **CLEAR SYNERGY** |
| --- | --- | --- | --- | --- | --- | --- |
| **2.1. Were participants aware of their assigned intervention during the trial?** | Y | N | N | N | N | N |
| **2.2. Were carers and people delivering the interventions aware of participants' assigned intervention during the trial?** | Y | N | N | N | N | N |
| **2.3. If Y/PY/NI to 2.1 or 2.2: Were important co-interventions balanced across intervention groups?** | Y |  |  |  |  |  |
| **2.4. Could failures in implementing the intervention have affected the outcome?** | Y | Y | Y | Y | Y | Y |
| **2.5. Did study participants adhere to the assigned intervention regimen?** | Y | Y | Y | Y | Y | Y |
| **2.6. If N/PN/NI to 2.3 or 2.5 or Y/PY/NI to 2.4: Was an appropriate analysis used to estimate the effect of adhering to the intervention?** | Y | Y | Y | Y | Y | Y |
| **Risk-of-bias judgement** | Some concern | Low | Low | Low | Low | Low |
| Optional: What is the predicted direction of bias due to deviations from intended interventions? | Favors experimental / Favors comparator / Toward null /Away from null / Unpredictable | Favors experimental / Favors comparator / Toward null /Away from null / Unpredictable | Favors experimental / Favors comparator / Toward null /Away from null / Unpredictable | Favors experimental / Favors comparator / Toward null /Away from null / Unpredictable | Favors experimental / Favors comparator / Toward null / Away from null / Unpredictable | Favors experimental / Favors comparator / Toward null / Away from null / Unpredictable |

Domain 3: Missing outcome data

| **Signaling questions** | **LoDoCo** | **COLCOT** | **COPS** | **LoDoCO2** | **CONVINCE** | **CLEAR SYNERGY** |
| --- | --- | --- | --- | --- | --- | --- |
| **3.1 Were data for this outcome available for all, or nearly all, participants randomized?** | Y | Y | Y | Y | Y | Y |
| **3.2 If N/PN/NI to 3.1: Is there evidence that result was not biased by missing outcome data?** |  |  |  |  |  |  |
| **3.3 If N/PN to 3.2: Could missingness in the outcome depend on its true value?** |  |  |  |  |  |  |
| **3.4 If Y/PY/NI to 3.3: Do the proportions of missing outcome data differ between intervention groups?** |  |  |  |  |  |  |
| **3.5 If Y/PY/NI to 3.3: Is it likely that missingness in the outcome depended on its true value?** |  |  |  |  |  |  |
| **Risk-of-bias judgement** | Low | Low | Low | Low | Low | Low |
| Optional: What is the predicted direction of bias due to missing outcome data? | Favors experimental / Favors comparator / Toward null /Away from null / Unpredictable | Favors experimental / Favors comparator / Toward null /Away from null / Unpredictable | Favors experimental / Favors comparator / Toward null /Away from null / Unpredictable | Favors experimental / Favors comparator / Toward null /Away from null / Unpredictable | Favors experimental / Favors comparator / Toward null / Away from null / Unpredictable | Favors experimental / Favors comparator / Toward null / Away from null / Unpredictable |

Domain 4: Risk of bias in measurement of the outcome

| **Signaling questions** | **LoDoCo** | **COLCOT** | **COPS** | **LoDoCo2** | **CONVINCE** | **CLEAR SYNERGY** |
| --- | --- | --- | --- | --- | --- | --- |
| **4.1 Was the method of measuring the outcome inappropriate?** | N | N | N | N | N | N |
| **4.2 Could measurement or ascertainment of the outcome have differed between intervention groups ?** | N | N | N | N | N | N |
| **4.3 If N/PN/NI to 4.1 and 4.2: Were outcome assessors aware of the intervention received by study participants ?** | N | N | N | N | N | N |
| **4.4 If Y/PY/NI to 4.3: Could assessment of the outcome have been influenced by knowledge of intervention received?** |  |  |  |  |  |  |
| **4.5 If Y/PY/NI to 4.4: Is it likely that assessment of the outcome was influenced by knowledge of intervention received?** |  |  |  |  |  |  |
| **Risk-of-bias judgement** | Low | Low | Low | Low | Low | Low |
| Optional: What is the predicted direction of bias in measurement of the outcome? | Favors experimental / Favors comparator / Toward null /Away from null / Unpredictable | Favors experimental / Favors comparator / Toward null /Away from null / Unpredictable | Favors experimental / Favors comparator / Toward null /Away from null / Unpredictable | Favors experimental / Favors comparator / Toward null /Away from null / Unpredictable | Favors experimental / Favors comparator / Toward null /Away from null / Unpredictable | Favors experimental / Favors comparator / Toward null /Away from null / Unpredictable |

Domain 5: Risk of bias in selection of the reported result

| **Signaling questions** | **LoDoCo** | **COLCOT** | **COPS** | **LoDoCo2** | **CONVINCE** | **CLEAR SYNERGY** |
| --- | --- | --- | --- | --- | --- | --- |
| **5.1 Was the trial analyzed in accordance with a pre-specified plan that was finalized before unblinded outcome data were available for analysis ?** | Y | Y | Y | Y | Y | Y |
| **Is the numerical result being assessed likely to have been selected, on the basis of the results, from...** |  |  |  |  |  |  |
| **5.2. ... multiple outcome measurements (e.g. scales, definitions, time points) within the outcome domain?** | N | N | N | N | N | N |
| **5.3 ... multiple analyses of the data?** | N | N | N | N | N | N |
| **Risk-of-bias judgement** | Low | Low | Low | Low | Low | Low |
| Optional: What is the predicted direction of bias due to selection of the reported result? | Favors experimental / Favors comparator / Toward null /Away from null / Unpredictable | Favors experimental / Favors comparator / Toward null /Away from null / Unpredictable | Favors experimental / Favors comparator / Toward null /Away from null / Unpredictable | Favors experimental / Favors comparator / Toward null /Away from null / Unpredictable | Favors experimental / Favors comparator / Toward null /Away from null / Unpredictable | Favors experimental / Favors comparator / Toward null /Away from null / Unpredictable |

Overall risk of bias

|  | **LoDoCo** | **COLCOT** | **COPS** | **LoDoCo2** | **CONVINCE** | **CLEAR SYNERGY** |
| --- | --- | --- | --- | --- | --- | --- |
| **Risk-of-bias judgement** | Some concern | Low | Low | Low | Low | Low |
| Optional: What is the predicted direction of bias due to selection of the reported result? | Favors experimental / Favors comparator / Toward null /Away from null / Unpredictable | Favors experimental / Favors comparator / Toward null /Away from null / Unpredictable | Favors experimental / Favors comparator / Toward null /Away from null / Unpredictable | Favors experimental / Favors comparator / Toward null /Away from null / Unpredictable | Favors experimental / Favors comparator / Toward null /Away from null / Unpredictable | Favors experimental / Favors comparator / Toward null /Away from null / Unpredictable |

**Sensitivity analysis:**

**Supplemental Figure S1.** Forest plot of primary efficacy outcomes without CONVINCE trial

**Supplemental Figure S2.** Forest plot of MACE (pre-COVID)

**Supplemental Figure S2.** Forest plot of acute coronary disease only


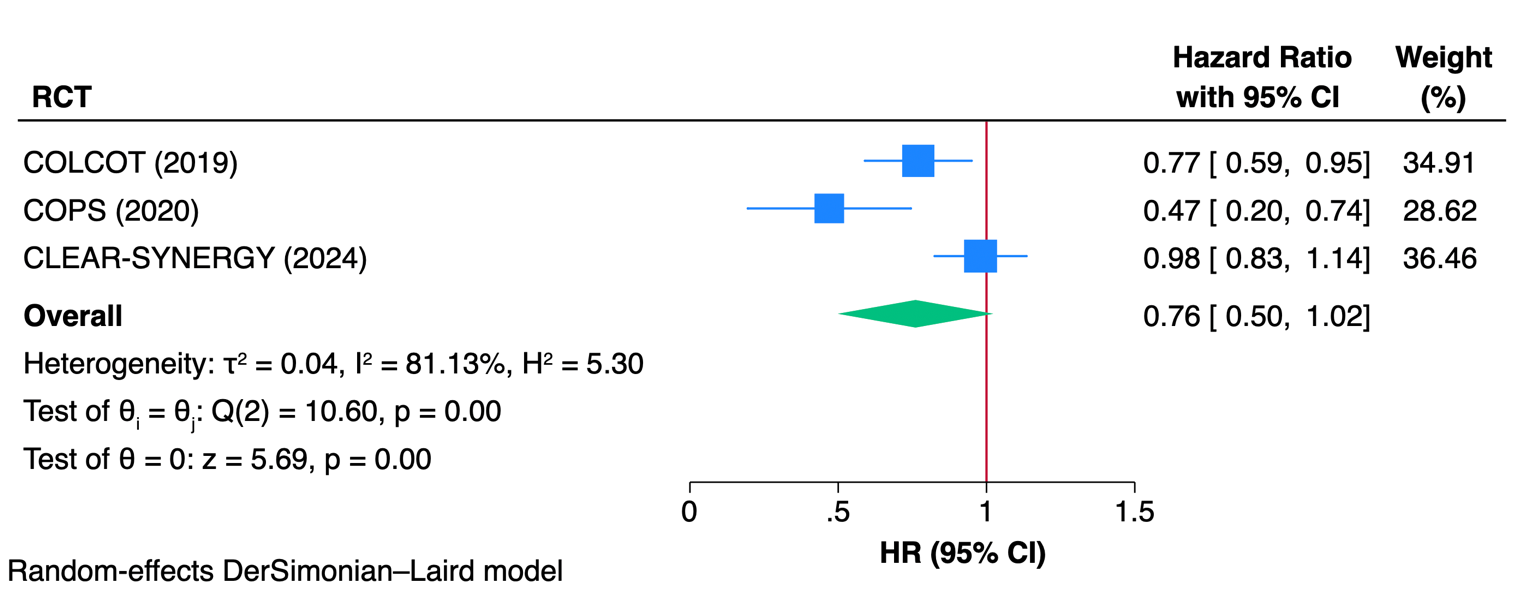


**Bias assessments**

**Supplemental Figure S4.** Funnel plots

| Endpoint | Funnel plot | Egger’s Test  P-value |
| --- | --- | --- |
| 1. MACE |  | p=0.24 |
| 1. Cardiovascular mortality |  | p=0.87 |
| 1. Myocardial infarction |  | p=0.72 |
| 1. Ischemic stroke |  | p=0.52 |
| 1. Urgent coronary revascularization |  | p=0.09 |
